# Coating matching recommendation based on improved fuzzy comprehensive evaluation and collaborative filtering algorithm

**DOI:** 10.1038/s41598-021-93628-4

**Published:** 2021-07-07

**Authors:** Yuan Xin, Bu Henan, Niu Jianmin, Yu Wenjuan, Zhou Honggen, Ji Xingyu, Ye Pengfei

**Affiliations:** 1 School of Mechanical Engineering, Jiangsu University of Science and Technology, Zhenjiang, 212100 Jiangsu China; 2 Shanghai Shipbuilding Technology Research Institute, Shanghai, 200032 China

**Keywords:** Engineering, Mathematics and computing

## Abstract

Coating matching design is one of the important parts of ship coating process design. The selection of coating matching is influenced by various factors such as marine corrosive environment, anti-corrosion period and working conditions. There are also differences in the coating performance requirements for different ship types and different coating parts. At present, the design of coating matching in shipyards depends on the experience of technologist, which is not conducive to the scientific management of ship painting process and the macro control of ship construction cost. Therefore, this paper proposes a hybrid algorithm of fuzzy comprehensive evaluation and collaborative filtering based on user label improvement (IFCE-CF). Based on the analytic hierarchy process (AHP), the evaluation index system of coating matching is constructed, and the weight calculation process of fuzzy comprehensive evaluation is optimized by introducing the user label weight. The collaborative filtering algorithm based on matrix decomposition is used to realize the accurate recommendation of coating matching. Historical coating process data of a shipyard between 2010 and 2020 are selected to verify the recommendation ability of the method in the paper. The results show that using the coating matching intelligent recommendation algorithm proposed in this paper, the root mean square error is < 1.02 and the mean absolute error is < 0.75, the prediction accuracy is significantly better than other research methods, which proves the effectiveness of the method.

## Introduction

Ship painting is one of the three pillars of modern shipbuilding processes and is used throughout the shipbuilding process^1,2^. As the anti-fouling barrier of the hull, the coating is essential for the structural integrity, hydrodynamic performance and service life of the ship^3^. The design of coating matching needs to consider many factors such as the anti-corrosion requirements of each part of the hull, the requirements of different coatings for surface treatment, and the matching system of coatings. It needs to be selected after technical negotiation between shipyard technologist and shipowners. The reasonable degree of coating is directly related to the construction cycle and cost of the ship as well as the service life and maintenance cycle of the ship^4^. With the development of the coating system, the manufacturers of coatings and the varieties of coating types are increasing, and new coating matching are emerging^5,6^. At the same time, there is an increasing demand for individualized coating matching design from ship owners. Reliable and accurate coating matching recommendation algorithm can provide technical guarantee for shipowners to select suitable coating matching. It is also a powerful tool to improve the core competitiveness of shipyards^7^.

The design of the coating matching is the design of the matching scheme of priming paint, intermediate paint and topcoat, including the design of the number of coating courses and dry film thickness, etc. As each part of the ship is in a different corrosive environment, the choice of coatings and the parameters of each coating process are also different. Traditionally, the selection of coatings relies on the experience of technologist and coating inventory, and there is no systematic statistics and analysis of the data related to coating matching. The increasing complexity of the ship's use environment has increased the requirements for anti-corrosion performance and reliability. The continuous development of coating technology has given rise to more alternative coating matching options. Therefore, designing an intelligent recommendation algorithm for coating matching is the way to meet the individual needs of ship owners and achieve scientific management of coating process.

Fuzzy comprehensive evaluation (FCE) is a nonlinear multi-objective comprehensive evaluation method, which can evaluate systems with fuzzy concepts. The method has been widely used in the fields of environmental assessment^8,9^, program decision-making^10^, risk assessment^11,12^, and system evaluation^13–15^. Jun Hu et al.^16^ established a fuzzy comprehensive evaluation system using seismicity rates, magnitude-frequency coefficients, reservoirs, regional geotectonic, stress fields and the spatial distribution of fracturing platforms as factors to quantitatively evaluate the seismic hazard of hydraulic fracturing area. Tong Si et al.^17^ constructed a multi-criteria comprehensive energy efficiency evaluation system including technical, environmental, economic and social benefits for the selection of control options for coal-fired pollutants. Haitao Ma et al.^18^ conducted a qualitative and quantitative assessment of the risks of multinational oil investments in Central Asia based on Delphi method and FCE. Yunna Wu et al.^19^ established a hierarchy to evaluate the benefits of waste-to-energy plants in terms of economic, environmental, and social benefits. Gang Chen et al.^20^ used throttle repeatability, speed tracking accuracy, speed repeatability, and driving shock as system evaluation indexes to evaluate the performance of unmanned robots. The above research results provide a good theoretical basis for the establishment of a fuzzy comprehensive evaluation system for coating matching.

Collaborative filtering (CF) algorithm is a category-based recommendation algorithm. It recommends items that may be of interest to the target user based on similarities between user groups or item groups. It plays an important role in the recommendation of many aspects such as social networks, movies, music, and articles^21–23^. Beiliang Cui et al.^24^ fused the similarity of user scores and the similarity of public choice terms into CF to achieve the prediction of the shareholding percentage of listed companies. Shun Li et al.^25^ proposed a personalized recommendation system based on CF to provide intelligent tariff recommendations for end users. Youness MADANI et al.^26^ used CF to recommend courses for learners. Arup Roy et al.^27^ proposed CF for changing customers in a restaurant recommendation system. All the above research results have achieved good recommendation results, but the application of intelligent recommendation algorithm represented by CF in the field of ship construction has been rarely reported.

At present, the transformation and upgrading of the shipbuilding industry driven by intelligent manufacturing is in its initial stage. Aiming at the problem that historical coating matching does not have a scientific and reasonable evaluation system, this paper proposes a fuzzy comprehensive evaluation method improved by user interest labels and analytic hierarchy process to construct the scoring matrix in the collaborative filtering algorithm to solve the cold start problem of collaborative filtering recommendation. And the collaborative filtering algorithm based on matrix decomposition is used to target the recommendation of coating matching to the target users, thus improving the prediction scoring accuracy of the traditional recommendation algorithm and realizing the effective recommendation of coating matching.

## Construction of fuzzy comprehensive evaluation system

FCE is a comprehensive evaluation method based on fuzzy mathematics and applying the principle of fuzzy relation synthesize theory to quantify some factors with unclear boundaries and not easy to quantify^28^. In this paper, FCE is used to build a coating matching evaluation system. At the same time, in order to consider the personalized coating customization needs of different ship owners, a fuzzy comprehensive evaluation method based on user label improvement is proposed.

### Construction of FCE index

The fuzzy comprehensive evaluation of coating matching is a complex decision-making process involving multiple factors and indexes. Therefore multiple interrelated and interacting evaluation indexes need to be considered in the evaluation process. By extensively collecting the relevant information of coating matching related research combined with the opinions of shipyard experts, the FCE index of coating matching is constructed and the set of coating matching evaluation index *U* is obtained.

1 $$ U = \{ u_{1} ,u_{2} , \ldots ,u_{m} \} $$

where *u*_*i*_ stands for each evaluation indicator; *m* is the total number of evaluation indicators.

**Figure 1:**
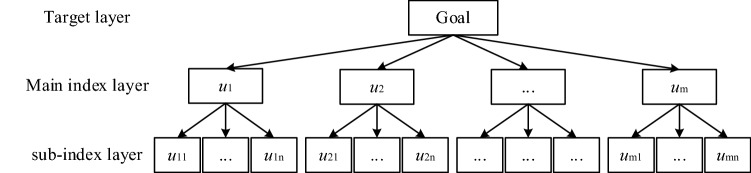
Hierarchical structure model.

In order to improve the scientificity and organization of the coating matching evaluation, the hierarchical structure of the evaluation indexes was divided using hierarchical analysis. The hierarchical analysis structure model of coating supporting evaluation includes: target layer, main index layer and sub-index layer, as shown in Fig. 1. The target layer is the overall goal to be achieved, which in this paper is the overall score of the coating matching evaluation. The main index layers are evaluation aspects importantly related to the evaluation of coating matching. The sub-index layer is a parent–child relationship with the main index layer, which is a further refinement and classification of the main index.

### Weight vector calculation based on hierarchical analysis and user labels

The determination of the weight of each evaluation index is the key to the fuzzy comprehensive evaluation of the coating matching. As a traditional method for determining the weight of evaluation indexes, hierarchical analysis is a decision-making method that uses a certain scale to objectively quantify human subjective judgments, on the basis of which qualitative and quantitative analysis is performed^29^. However, the index weights determined based on the hierarchical analysis method only reflect the objective understanding of the shipyard experts on each evaluation index of the coating matching. When choosing the actual coating matching, shipowners are also influenced by their own economic conditions, expected protection time of the coating and other subjective individual needs. It is not possible to fully unify with the shipyard experts' opinions.

To this end, this paper introduces the weights of user labels while using hierarchical analysis to determine the weights of each major index and sub-index. The evaluation indexes of the main index layer are used as user-selectable labels.

#### Construction of the judgment matrix

The construction of the judgment matrix is a prerequisite for determining the index weights using the hierarchical analysis method. On the basis of establishing the hierarchical analysis model, the elements of each layer are compared two by two to construct the judgment matrix $C = (C_{{ij}} )_{{n \times n}}$^30^, as shown in Eq. (2).

2 $$ C = \left[ {\begin{array}{*{20}c}    {C_{{11}} } & {C_{{12}} } &  \cdots  & {C_{{1n}} }  \\    {C_{{21}} } & {C_{{22}} } &  \cdots  & {C_{{2n}} }  \\     \vdots  &  \vdots  &  \vdots  &  \vdots   \\    {C_{{n1}} } & {C_{{n2}} } &  \cdots  & {C_{{nn}} }  \\   \end{array} } \right] $$

where $C_{{ij}}  > 0$, $C_{{ij}}  = 1/C_{{ji}} (i \ne j)$, $C_{{ii}}  = 1\;(i,j = 1,2, \ldots ,n)$. $C_{{ij}}$ represents the relative importance of factor *i* when compared with factor *j*, and *n* is the number of indexes in each layer.

#### Judgment matrix consistency test

The consistency test of the judgment matrix was performed to ensure the reasonableness of the evaluation results^31^. First, the consistency index (*CI*), which measures the negative average of the remaining eigenvalues of the judgment matrix other than the maximum eigenvalue, is calculated using Eq. (3).

3 $$ CI = \frac{{\lambda _{{\max }}  - n}}{{n - 1}} $$

where *λ*_max_ represents the maximum characteristic root of the judgment matrix; *n* is the order of the judgment matrix.

Next, the random consistency ratio (*CR*), which measures whether the judgment matrix has satisfactory consistency, is calculated using Eq. (4).

4 $$ CR = \frac{{CI}}{{RI}} $$

where *RI* is the average random consistency index, and the values of *RI* are shown in Table 1. When $CR < 0.10$, the judgment matrix has satisfactory consistency.

**Table 1:** Average random consistency index.

Matrix order	1	2	3	4	5	6	7	8	9
*RI*	0.00	0.00	0.58	0.90	1.12	1.24	1.32	1.41	1.45

#### Calculation of the weight vector

After the judgment matrix passes the consistency test, the index weights based on hierarchical analysis are obtained by solving the eigenvalues of the judgment matrix. Methods for calculating the characteristic value of the judgment matrix include square root method, characteristic root method, least-square method, etc. In this paper, the summation method used in the literature^32^ is used for the solution of the characteristic vectors of the judgment matrix, as shown in Eq. (5).

5 $$ W_{i}  = \frac{1}{n}\sum\limits_{{j = 1}}^{n} {\frac{{C_{{ij}} }}{{\sum\nolimits_{{k = 1}}^{n} {C_{{kj}} } }}} ,\quad i = 1,2, \ldots ,n $$

The steps for solving *W*_*i*_ are: (1) normalize the factors of each column of the judgment matrix *C*; (2) add the normalized factors of each column; (3) divide each element of the summed vector by *n* to obtain the weight vector.

Labels are used to describe item characteristics. Due to the individual needs of users, the level of agreement with expert rating metrics varies from user to user. Therefore, this paper introduces the weight $W_{U}$ of user labels, and uses the evaluation indexes of the main index layer as the labels that can be selected by users.

6 $$ W_{U}  = [U_{1} ,U_{2} , \ldots ,U_{\varepsilon } ] $$

where $U_{i}$ is the weight of each label; $\varepsilon$ is the number of labels, and $\sum\nolimits_{{i = 1}}^{\varepsilon } {U_{i}  = 1}$.

The weight vector $W$ of the combined user labels is:

7 $$ W = \alpha _{1} W_{1}  + \alpha _{{\text{2}}} W_{U} {\text{ = [}}W_{1} ,W_{2} {\text{,}} \ldots {\text{,}}W_{\varepsilon } {\text{]}} $$

where $W_{1}$ is the characteristic vector calculated from the judgment matrix of the main index layer; $\alpha _{1}$, $\alpha _{2}$ are the influence factors of main index layer and user label respectively. $\alpha _{2}  = 1 - \alpha _{1}$, the value of $\alpha _{2}$ reflects the importance of the user's opinion. However, when the value of $\alpha _{2}$ is too large, it will affect the reasonableness of the coating matching evaluation results. Therefore, it is necessary to combine the opinions of shipyard painting experts to take the values of $\alpha _{1}$, $\alpha _{2}$.

Using the main index layer weight vector *W* and the subindex weight vector $W_{{ij}}$, the composite score vector $W_{c}$ is obtained.

8 $$ W_{c}  = \left[ {W_{{c1}} ,W_{{c2}} , \ldots ,W_{{c\rho }} } \right] $$

where $$W_{{ci}}  = W_{i}  \times W_{{ij}}$$.

### Judgment matrix creation

The evaluation set is a collection of the total evaluation results that the evaluator can make about the evaluation object. The values of the evaluation set *V* and the standard values are determined by the domain experts according to the characteristics of the evaluation object.

9 $$ V = \left\{ {v_{1} ,v_{2} , \ldots ,v_{x} } \right\} $$

where $v_{i}$ represents the *i*-th evaluation result; *x* is the total number of evaluation elements.

Single-index evaluation of each evaluation index in the evaluation set *V* is performed to obtain the single-factor evaluation set of the *i*-th index.

10 $$ r_{i}  = \left( {r_{{i1}} ,r_{{i2}} , \ldots ,r_{{in}} } \right) $$

where $r_{{ij}}$ represents the degree of membership of the *i*-th index $u_{i}$ in the index set *U* corresponding to the *j*-th element $v_{j}$ in the evaluation set *V*.

Following that, the total judgment matrix *R* is constructed.

11 $$ R = \left[ {\begin{array}{*{20}c}    {r_{1} }  \\    {r_{2} }  \\     \vdots   \\    {r_{n} }  \\   \end{array} } \right] = \left[ {\begin{array}{*{20}c}    {r_{{11}} } & {r_{{12}} } &  \cdots  & {r_{{1n}} }  \\    {r_{{21}} } & {r_{{22}} } &  \cdots  & {r_{{2n}} }  \\     \vdots  &  \vdots  &  \vdots  &  \vdots   \\    {r_{{n1}} } & {r_{{n2}} } &  \cdots  & {r_{{nn}} }  \\   \end{array} } \right] $$

### Multi-level fuzzy comprehensive evaluation

FCE includes single-level fuzzy comprehensive evaluation (SFCE) and multi-level fuzzy comprehensive evaluation (MFCE). MFCE is suitable for evaluation problems with more evaluation factors and evaluation objectives with fuzzy characteristics. In order to obtain comprehensive evaluation results of the coating matching, MFCE is used in this paper.

The fuzzy comprehensive evaluation vector is obtained by fuzzy transforming the evaluation matrix *R* with the comprehensive weighting vector $W_{c}$. The fuzzy integrated evaluation vector *B* represents the final result of the coating matching evaluation based on the fuzzy algorithm.

12 $$ B = W_{c}  \circ R = \left( {b_{1} ,b_{2} , \ldots ,b_{n} } \right) $$

where $\circ$ represents the fuzzy operator, common operator models include: maximum judgment model $M( \wedge , \vee )$, weighted average model $M( \cdot , \vee )$, fuzzy vector model $M( \cdot , \oplus )$.Since each index affects the evaluation of coating matching, the $M( \cdot , \vee )$ model is used for fuzzy transformation in this paper. The solution of the factor in vector *B* is as in Eq. (13).

13 $$ b_{j}  = \mathop  \vee \limits_{{i = 1}}^{m} \left( {W_{{ci}} ,r_{{ij}} } \right) = \mathop {\max }\limits_{{1 \le i \le m}} \{ W_{{ci}} ,r_{{ij}} \} ,j = 1,2, \ldots ,n $$

## Collaborative filtering recommendation algorithm

Collaborative filtering recommendation is a recommendation method based on user ratings of items. It includes based on neighborhood approach and based on latent factor model approach^33^. The collaborative filtering algorithm based on matrix decomposition is one of the methods based on the latent factor model, which has better results in solving the data sparsity problem. The matrix decomposition model maps users and items to the same latent semantic space, and explains ratings by characterizing users and items on factors.

**Figure 2:**
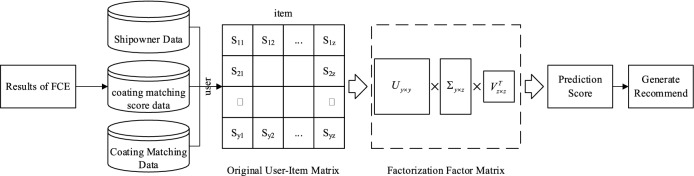
Recommended process for coating matching.

Due to the incomplete historical order data of the shipyard, there are a lot of missing data in the scoring matrix. In order to avoid the impact of missing data on coating matching recommendation, this paper adopts a collaborative filtering algorithm based on matrix decomposition for the design of coating matching recommendation system, and the process is shown in Fig. 2. Firstly, the shipowner-coating matching scoring matrix is constructed based on the results of fuzzy comprehensive evaluation of coating matching; Then, singular value decomposition technology is used to decompose the scoring matrix; Finally, the recommendation of coating matching is realized by training the predictive scoring model.

### Establishment of scoring matrix

Unlike recommendations of movies, books, etc., there is currently no evaluation score from shipyards for coating matching that have been used. Therefore, this paper adopts the fuzzy comprehensive evaluation method based on user labels improvement to obtain the coating matching score, and builds the shipowner-coating matching scoring matrix $S_{{y \times z}}$ based on this.

Where *y* represents the number of shipowners, *z* represents the number of coating matching, and $S_{{ij}}$ represents the score of coating matching *j* used by shipowner *i*.

### Matrix decomposition

Matrix decomposition can represent the original scoring matrix as a new easy-to-handle form. Singular value decomposition algorithm is a commonly used matrix decomposition algorithm to reduce the dimensionality of data. The principle of singular value decomposition is to decompose the initial scoring matrix *S* into the form of multiplication of three matrices *U*, $\Sigma$, $V^{T}$^34^.

14 $$ S = U \times \Sigma  \times V^{T} $$

where *U* and $V^{T}$ are two unitary matrices of $y \times y$ and $z \times z$, respectively, *U* represents the user's preference for the item, and $V^{T}$ represents the similarity between the item and the underlying factors. $\Sigma$ is a nonsingular matrix of $y \times z$ with all elements zero except for the diagonal elements.

15 $$ \Sigma  = diag\left( {\sigma _{1} ,\sigma _{2} , \ldots ,\sigma _{y} } \right) $$

where $\sigma _{i}$ is the singular value, $\sigma _{i}  > 0\;(i = 1,2, \ldots ,y)$; *y* is the rank of the matrix *S*.

Usually the sum of the first *k* singular values occupies more than 90% of the total sum of singular values, so the eigenvectors corresponding to the first *k* singular values are used to describe the original scoring matrix. Reducing the dimensionality of the original scoring matrix while retaining most of the information in the original matrix. The dimensionality reduction formula of singular value decomposition is shown in Eq. (16).

16 $$ S = U_{{y \times y}}  \times \Sigma _{{y \times z}}  \times V_{{z \times z}}^{T}  \approx U_{{y \times k}}  \times \Sigma _{{k \times k}}  \times V_{{k \times z}}^{T} $$

where $$k < z$$.

### Prediction scoring model

The collaborative filtering algorithm based on singular value decomposition decomposes the original data matrix into matrices $p_{u}$ and $q_{i}$ by training on user rating information when predicting ratings. $p_{{u(i)}}  = (i_{1} ,i_{2} , \ldots ,i_{k} )^{T}$ is a column in $p_{u}$ that represents the user's potential interest metric; $q_{{i(j)}}  = (j_{1} ,j_{2} , \ldots ,j_{k} )^{T}$ is a column in $q_{i}$ that represents the attribute metric of the item; *k* is the number of latent factors set in the matrix decomposition process. The singular value decomposition score prediction model is shown in Eq. (17).

17 $$ \mathop r\limits^{ \wedge } _{{u,i}}  = \mu  + b_{i}  + b_{u}  + q_{i} ^{T} p_{u} $$

where $\mu$ is the overall average rating; $b_{u}$, $b_{i}$ represent the deviation of user *u* and item *i* from the average rating, respectively; $q_{i} ^{T} p_{u}$ represents the overall interest level of the user in the item.

In the training process of the matrix decomposition model, the error between predicted and true scores is first calculated based on the scoring model.

18 $$ e_{{u,i}} \mathop  = \limits^{{def}} r_{{u,i}}  - \mathop r\limits^{ \wedge } _{{u,i}} $$

where $r_{{u,i}}$ is the actual rating of item *i* by user *u*.

The loss function uses the sum of squares to calculate the error, which is a statistical way of the error. Its magnitude reflects the difference between the predicted score and the true score. However, an undersize loss function can lead to overfitting of the trained model with the training set data and the generalization performance of the model is reduced. The overfitting phenomenon is prevented by introducing a regularization constraint term^35^. In this paper, L2 regularization is introduced to prevent overfitting and L1 regularization is introduced to enhance feature selection^36^. The loss function after introducing the regularization parameter is shown in Eq. (19).

19 $$ \mathop {\min }\limits_{{b_{ * } ,q_{ * } ,p_{ * } }} \sum\limits_{{(u,i) \in \kappa }} {e_{{u,i}}^{2} }  + \lambda _{1} \left( {b_{i} ^{2}  + b_{u}^{2}  + ||q_{i} ||_{1}  + ||p_{u} ||_{1} } \right) + \lambda _{2} \left( {b_{i} ^{2}  + b_{u}^{2}  + ||q_{i} ||^{2}  + ||p_{u} ||^{2} } \right) $$

where $\lambda _{1}$ is the L1 regularization factor, $\lambda _{2}$ is the L2 regularization factor; $\kappa  = \{ (u,i):r_{{u,i}} known\}$.

In this paper, stochastic gradient descent is used to update the parameters in the training set^34^, and the parameters are updated as shown in Eq. (20).

20 $$ \left\{ {\begin{array}{*{20}l}    {b_{u}  \leftarrow b_{u}  + \gamma  \cdot \left( {e_{{u,i}}  - \lambda _{1}  \cdot b_{u}  - \lambda _{2}  \cdot b_{u} } \right)} \hfill  \\    {b_{i}  \leftarrow b_{i}  + \gamma  \cdot \left( {e_{{u,i}}  - \lambda _{1}  \cdot b_{i}  - \lambda _{2}  \cdot b_{i} } \right)} \hfill  \\    {q_{i}  \leftarrow q_{i}  + \gamma  \cdot \left( {e_{{u,i}}  \cdot p_{u}  - \lambda _{1}  \cdot q_{i}  - \lambda _{2}  \cdot q_{i} } \right)} \hfill  \\    {p_{u}  \leftarrow p_{u}  + \gamma  \cdot \left( {e_{{u,i}}  \cdot q_{i}  - \lambda _{1}  \cdot p_{u}  - \lambda _{2}  \cdot p_{u} } \right)} \hfill  \\   \end{array} } \right. $$

where $\gamma$ is the learning rate, which is used to control the rate of gradient descent.

The trained parameters are used in the predictive scoring model. When a user is entered, the system makes a rating prediction. The coating matching are then recommended in order of the rating.

### A hybrid algorithm of fuzzy comprehensive evaluation and collaborative filtering based on improved user labeling

**Figure 3:**
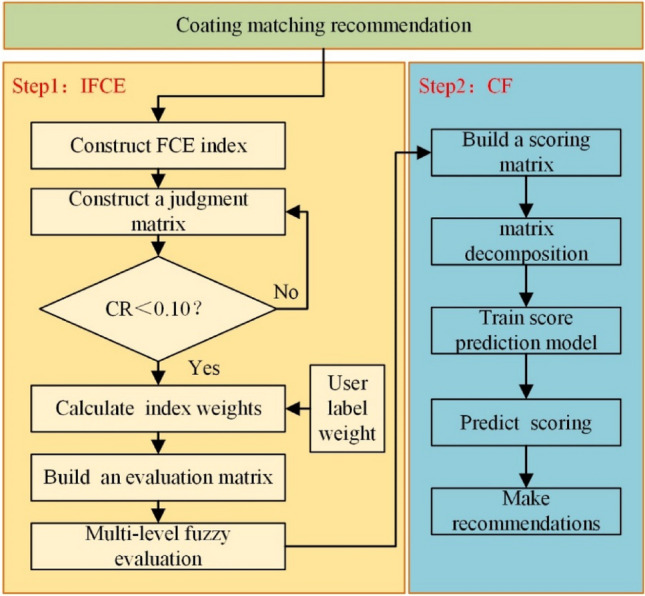
Algorithm flow chart.

The flow of the improved fuzzy comprehensive evaluation and collaborative filtering hybrid algorithm based on user labels is shown in Fig. 3, and its computational steps are as follows:

1. Construct fuzzy comprehensive evaluation index;
2. Construct the judgment matrix, and judge whether the matrix passes the consistency test. Reconstruct the judgment matrix if it does not meet the consistency requirements;
3. Calculate the weights of each evaluation index based on the judgment matrix. The weights of user labels are introduced and weighted to obtain the final weights occupied by each index;
4. Determine the evaluation set of fuzzy comprehensive evaluation and determine the evaluation matrix;
5. Perform multi-level fuzzy comprehensive evaluation to get the final evaluation results of coating matching;
6. Construct the shipowner-coating matching scoring matrix based on the fuzzy comprehensive evaluation results of the coating matching;
7. Decompose the scoring matrix using singular value decomposition technology;
8. Train predictive scoring model to realize coating matching recommendation.

## Analysis of prediction results and comparison of models

Different parts of the hull have different anti-corrosion requirements and the coatings used are very different. This leads to differences in the evaluation indexes. For this reason, this article uses the flat bottom as an example to verify the matching recommended effects of priming paint, intermediate paint and topcoat.

### Fuzzy comprehensive evaluation of coating matching

#### Evaluation index structure model

**Figure 4:**
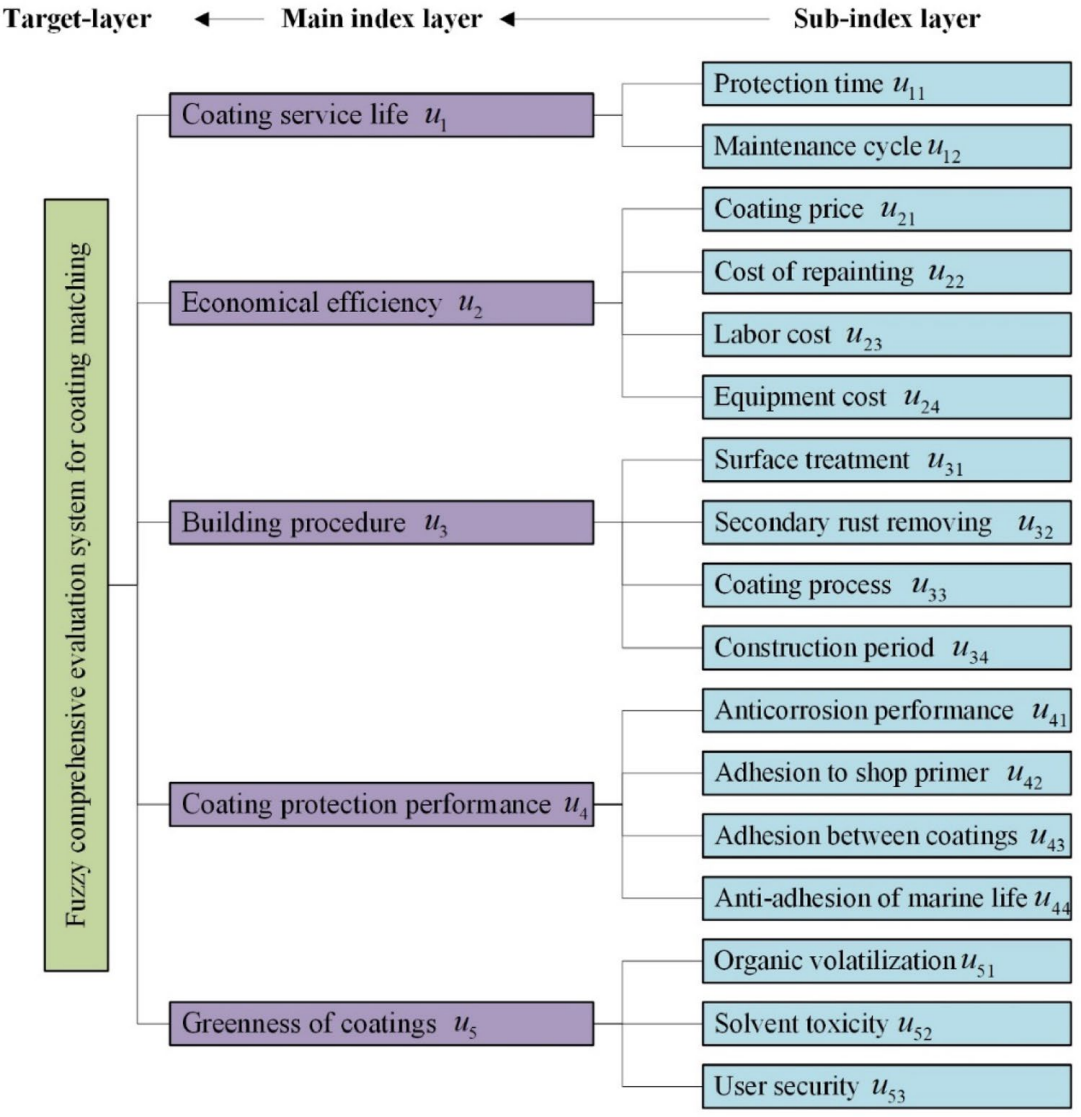
Structure of evaluation indexes for coating matching on flat bottom part.

The pros and cons of the matching coating are closely related to the actual service life of the coating, and the service life of the coating directly reflects the quality of the coating effect. However, the actual design is also influenced by the shipowner's consideration at the economic level, the actual level of craftsmanship of the shipyard's coating construction, and the coating protection performance of different parts of the hull. In addition, with the increasing awareness of environmental protection, green and environmentally friendly paints are more likely to be favored by the majority of shipowners. Therefore, the evaluation of the coating matching should take into account the five main indicators of coating service life, economical efficiency, building procedure, coating protection performance and greenness of coatings. This paper extracts the main indexes and sub-indexes for the evaluation of the coating matching on the flat bottom part of the ship based on the production practice experience and communication with the experts at the ship painting site. The indexes identified through screening are shown in Fig. 4.

#### Calculation of evaluation index weights

By inviting experts in the field of ship painting to compare each index of the coating matching, a judgment matrix *C* is established. The results of weight calculation for all main indexes and sub-indexes were tested for consistency. As shown in Tables 2, 3, 4, 5, 6 and 7, they are the main index layer judgment matrix, coating service life judgment matrix, economical efficiency judgment matrix, building procedure judgment matrix, coating protection performance judgment matrix and greenness of coatings judgment matrix, respectively.

**Table 2:** The main index layer judgment matrix.

G	*u*_1_	*u*_2_	*u*_3_	*u*_4_	*u*_5_	Weight	Consistency test
*u*_1_	1	2	3	1	2	0.2976	*λ*_max_ = 5.0133
*u*_2_	1/2	1	2	1/2	1	0.1579	*CI* = 0.0033
*u*_3_	1/3	1/2	1	1/3	1/2	0.089	*CR* = 0.003 < 0.1
*u*_4_	1	2	3	1	2	0.2976	
*u*_5_	1/2	1	2	1/2	1	0.1579

**Table 3:** Coating service life judgment matrix.

*u*_1_	*C*_1_	*C*_2_	Weight
*C*_1_	1	4	0.8
*C*_2_	1/4	1	0.2

**Table 4:** Economical efficiency judgment matrix.

*u*_2_	*C*_1_	*C*_2_	*C*_3_	*C*_4_	Weight	Consistency test
*C*_1_	1	6	5	3	0.5577	*λ*_max_ = 4.0788
*C*_2_	1/6	1	1/2	1/4	0.0705	*CI* = 0.0263
*C*_3_	1/5	2	1	1/3	0.1124	*CR* = 0.0295 < 0.1
*C*_4_	1/3	4	3	1	0.2594

**Table 5:** Building procedure judgment matrix.

*u*_3_	*C*_1_	*C*_2_	*C*_3_	*C*_4_	Weight	Consistency test
*C*_1_	1	1	1/2	1/3	0.1411	*λ*_max_ = 4.0104
*C*_2_	1	1	1/2	1/3	0.1411	*CI* = 0.0035
*C*_3_	2	2	1	1/2	0.2631	*CR* = 0.0039 < 0.1
*C*_4_	3	3	2	1	0.4547

**Table 6:** Coating protection performance judgment matrix.

*u*_4_	*C*_1_	*C*_2_	*C*_3_	*C*_4_	Weight	Consistency test
*C*_1_	1	1/2	1/2	1	0.1667	*λ*_max_ = 4
*C*_2_	2	1	1	2	0.3333	*CI* = 0
*C*_3_	2	1	1	2	0.3333	*CR* = 0 < 0.1
*C*_4_	1	1/2	1/2	1	0.1667

**Table 7:** Greenness of coatings judgment matrix.

*u*_5_	*C*_1_	*C*_2_	*C*_3_	Weight	Consistency test
*C*_1_	1	1/2	1	0.25	*λ*_max_ = 3
*C*_2_	2	1	2	0.5	*CI* = 0
*C*_3_	1	1/2	1	0.25	*CR* = 0 < 0.1

The user interest tags used in this article are: $tag_{1}$-coating service life, $tag_{2}$-economical efficiency, $tag_{3}$-building procedure, $tag_{4}$-coating protection performance, $tag_{5}$-greenness of coatings. Different shipowners make the choice of labels and the setting of label weights according to their individual requirements. In this paper, we take a shipyard's H1127/7 type vessel as an example, and the label weights set by the shipowner are shown in Table 8.

**Table 8:** Label weights selected by shipowners.

Label	*tag*_1_	*tag*_2_	*tag*_3_	*tag*_4_	*tag*_5_
Weight	0.35	0.1	0.15	0.2	0.2

In Eq. (7), set $\alpha _{1}  = 0.7$, $\alpha _{2}  = 0.3$ according to the opinion of ship painting experts.

21 $$ W = 0.7W_{1}  + 0.3W_{2}  = [0.314,0.14,0.107,0.269,0.17] $$

From Eqs. (8) and (21), the comprehensive score vector is calculated as:

22 $$ \begin{aligned}   W_{c}  &  = [0.251,0.062,0.078,0.011,0.016,0.036,0.015,0.015,0.028, \\     & \quad 0.049,0.045,0.089,0.089,0.045,0.043,0.085,0.043] \\  \end{aligned} $$

#### Fuzzy comprehensive evaluation result

The common coating matching schemes for the flat bottom part obtained from the survey of shipyards are shown in Table 9.

**Table 9:** Common coating matching for flat bottom parts.

Coating name	Matching system	Number of spraying	Thickness of dry film/μm	Thickness of wet film/μm	Solid content/%	Painting interval (23℃)
**1st set**						
Priming paint	Asphalt-based antirust paint	3	50	79	56	24H/30D
Intermediate paint	–	–	–	–	–	–
Topcoat	Asphalt-based anti-fouling paint	2	50	79	60	24H/36D
**2nd set**						
Priming paint	Chlorinated rubber antirust paint	2	40	87	46	8H/24D
Intermediate paint	–	–	–	–	–	–
Topcoat	Anti-fouling paint of chlorinated rubber	2	40	120	34	8H/24D
**⋮**						
**11th set**						
Priming paint	Epoxy asphalt antirust paint	2	50	79	56	24H/30D
Intermediate paint	Chloride rubber aluminum powder thick paste type antirust paint	2	35	73	46	8H/24D
Topcoat	Acrylic long-lasting anti-fouling paint	2	100	133	46	12H/24D

The evaluation matrix determined by experts in the field of ship painting is as follows:

23 $$ R = \left[ {\begin{array}{*{20}c}    {0.51} & {0.62} & {0.91} & {0.52} & {0.71} & {0.78} & {0.89} & {0.55} & {0.81} & {0.82} & {0.78}  \\    {0.53} & {0.65} & {0.89} & {0.49} & {0.7} & {0.75} & {0.91} & {0.56} & {0.78} & {0.79} & {0.81}  \\    {0.91} & {0.71} & {0.85} & {0.61} & {0.84} & {0.54} & {0.56} & {0.81} & {0.8} & {0.78} & {0.75}  \\    {0.71} & {0.73} & {0.87} & {0.69} & {0.92} & {0.87} & {0.88} & {0.87} & {0.84} & {0.86} & {0.85}  \\    {0.61} & {0.71} & {0.81} & {0.69} & {0.79} & {0.82} & {0.8} & {0.61} & {0.63} & {0.59} & {0.6}  \\    {0.92} & {0.91} & {0.89} & {0.9} & {0.89} & {0.88} & {0.9} & {0.89} & {0.87} & {0.91} & {0.92}  \\    {0.71} & {0.91} & {0.61} & {0.69} & {0.92} & {0.61} & {0.61} & {0.81} & {0.75} & {0.72} & {0.71}  \\    {0.69} & {0.9} & {0.62} & {0.7} & {0.91} & {0.59} & {0.62} & {0.8} & {0.74} & {0.69} & {0.69}  \\    {0.71} & {0.91} & {0.6} & {0.61} & {0.91} & {0.91} & {0.71} & {0.81} & {0.75} & {0.7} & {0.71}  \\    {0.72} & {0.9} & {0.62} & {0.59} & {0.89} & {0.9} & {0.72} & {0.8} & {0.74} & {0.67} & {0.73}  \\    {0.71} & {0.9} & {0.92} & {0.72} & {0.89} & {0.89} & {0.92} & {0.72} & {0.92} & {0.92} & {0.92}  \\    {0.9} & {0.89} & {0.91} & {0.88} & {0.9} & {0.91} & {0.92} & {0.88} & {0.91} & {0.91} & {0.91}  \\    {0.95} & {0.95} & {0.8} & {0.85} & {0.91} & {0.81} & {0.94} & {0.81} & {0.79} & {0.78} & {0.8}  \\    {0.61} & {0.62} & {0.91} & {0.59} & {0.91} & {0.89} & {0.88} & {0.89} & {0.65} & {0.65} & {0.89}  \\    {0.84} & {0.75} & {0.86} & {0.83} & {0.86} & {0.59} & {0.86} & {0.84} & {0.82} & {0.83} & {0.68}  \\    {0.84} & {0.82} & {0.69} & {0.8} & {0.72} & {0.69} & {0.68} & {0.69} & {0.85} & {0.85} & {0.75}  \\    {0.59} & {0.74} & {0.68} & {0.82} & {0.68} & {0.91} & {0.9} & {0.68} & {0.63} & {0.62} & {0.65}  \\   \end{array} } \right] $$

The comprehensive evaluation result *B* is:

24 $$ \begin{aligned}   B &  = W_{C}  \circ R = [0.128,0.156,0.228,0.13, \\     & \quad 0.178,0.196,0.223,0.138,0.203,0.206,0.196] \\  \end{aligned} $$

### Collaborative filtering recommendation results and model comparison

#### Data set

A total of 1200 painting history data from a shipyard between 2010 and 2020 are selected to verify the recommendation ability of the method in the paper. Among them, 1000 are used as the training set and 200 as the test set. The results of the partial coating matching scores are shown in Table 10.

**Table 10:** Partial coating matching scoring results.

No.	Ship owner A	Ship owner B	Ship owner C	Ship owner D	Ship owner E	Ship owner F	Ship owner G
Coating matching 1	0.128	0.11	0.128	0.122	–	0.11	0.097
Coating matching 2	0.156	0.133	–	0.148	–	0.133	0.118
Coating matching 3	0.228	–	–	0.217	0.217	–	–
Coating matching 4	0.13	0.112	0.13	–	0.124	0.112	–
Coating matching 5	0.178	–	–	–	0.17	–	0.136
Coating matching 6	0.196	–	0.196	–	–	0.168	0.149
Coating matching 7	0.223	–	0.223	0.223	–	–	–
Coating matching 8	0.138	0.118	0.138	0.138	0.138	–	–
Coating matching 9	0.203	–	–	–	–	0.174	0.155
Coating matching 10	0.206	–	–	–	0.196	0.176	–
Coating matching 11	0.196	0.168	–	–	0.186	0.168	0.149

#### Evaluation index

The core idea of the recommended performance metrics is to compare the difference between the recommended result data and the actual data. Mean absolute error (MAE) and root mean square error (RMSE) are used to measure the performance of recommendation algorithm^37^. MAE measures the absolute deviation between the user's real score and the actual score. The smaller the MAE is, the higher the accuracy of the prediction score is. RMSE reflects the degree of deviation between the predicted score and the actual score. The square operation is carried out before the error summation, which increases the punishment for the prediction error user score. The lower the RMSE is, the higher the accuracy of the prediction score is. The calculation formula is as follows:

25 $$ {\text{MAE}} = \frac{{\sum {_{{(u,i) \in T}} \left| {\mathop r\limits^{ \wedge } _{{u,i}}  - r_{{u,i}} } \right|} }}{{\left| T \right|}} $$

26 $$ {\text{RMSE}} = \sqrt {\frac{{\sum {_{{(u,i) \in T}} (\mathop r\limits^{ \wedge } _{{u,i}}  - r_{{u,i}} )^{2} } }}{{\left| T \right|}}} $$

where $r_{{u,i}}$ is the actual score of item *i* by user *u*; $\mathop r\limits^{ \wedge } _{{u,i}}$ is the predicted score of item *i* by user *u*; *T* represents the test set.

#### Recommended results analysis

After obtaining reasonable coating matching evaluation results, the accuracy of the recommended coating matching results obtained by the ship owner depends on the training of the parameters of the collaborative filtering algorithm. In this paper, the collaborative filtering algorithm is trained based on the coating matching data from shipyards. The parameters of collaborative filtering are set and adjusted to obtain better recommendation accuracy.

**Figure 5:**
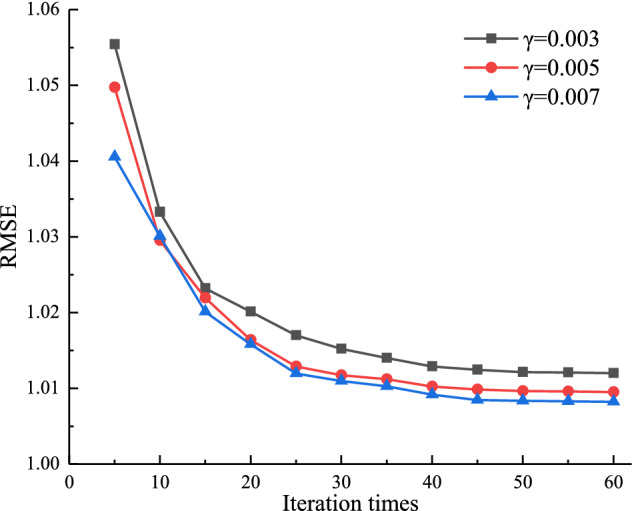
The influence of learning rate *γ* on the algorithm of this paper.

In order to analyze the effect of learning rate *γ* on the algorithm in this paper, let the number of latent factors *k* = 20, the L1 regularization factor *λ*_1_ = 0.2, the L2 regularization factor *λ*_2_ = 0.2, the number of iterations *δ* ∈ [5, 60]; the learning rate *γ* be 0.003, 0.005, 0.007, respectively. The results of learning rate optimization are shown in Fig. 5. From the figure, it can be seen that the RMSE value decreases gradually with the increase of the number of iterations, and the RMSE value decreases rapidly when the number of iterations *δ* < 25. When *δ* > 25, the RMSE value changes slowly and smoothly. The final RMSE of the algorithm is minimized for *γ* = 0.007.

**Figure 6:**
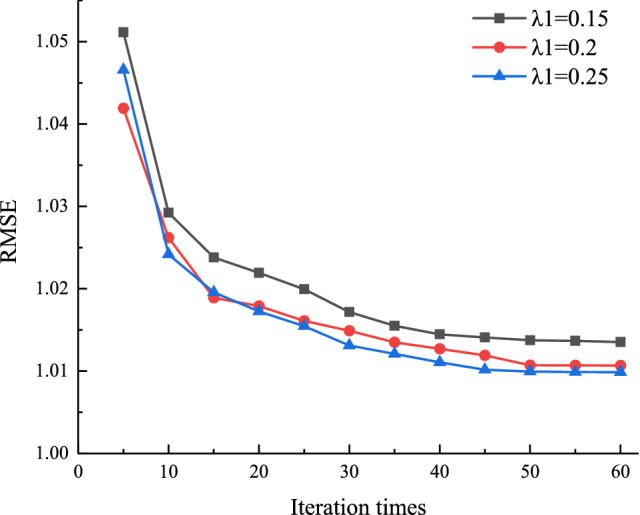
The influence of L1 regularization factor *λ*_1_ on the algorithm of this paper.

In order to analyze the effect of L1 regularization factor *λ*_1_ on the algorithm in this paper, let the number of latent factors *k* = 20, the L2 regularization factor *λ*_2_ = 0.2, the learning rate *γ* = 0.007, the number of iterations $\delta  \in [5,60]$; the L1 regularization factor *λ*_1_ is taken as 0.15, 0.2, and 0.25, respectively. The results of the regularization parameter optimization are shown in Fig. 6. It can be seen from the figure that the value of RMSE gradually decreases as the number of iterations increases. When the number of iterations *δ* > 50, the RMSE value does not change significantly. When *λ*_1_ = 0.25, the final RMSE value is the lowest.

**Figure 7:**
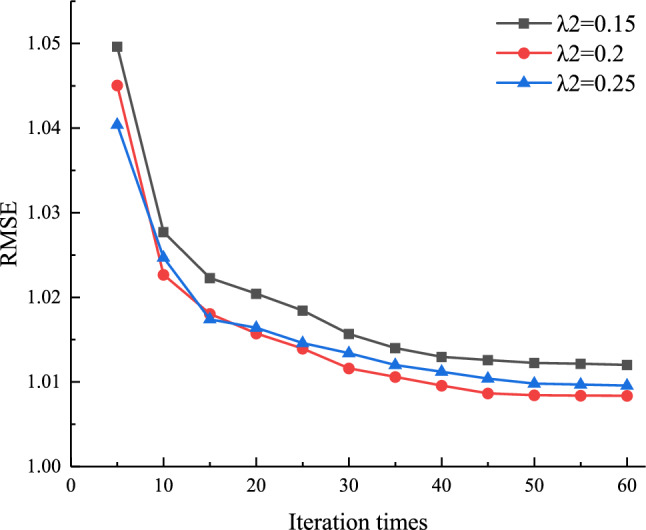
The influence of L2 regularization factor *λ*_2_ on the algorithm of this paper.

In order to analyze the effect of L2 regularization factor *λ*_2_ on the algorithm in this paper, let the number of latent factors *k* = 20, the L1 regularization factor *λ*_1_ = 0.25, the learning rate *γ* = 0.007, the number of iterations $\delta  \in [5,60]$; the L2 regularization factor *λ*_2_ is taken as 0.15, 0.2, and 0.25, respectively. The results of the regularization parameter optimization are shown in Fig. 7. It can be seen from the figure that the value of RMSE gradually decreases as the number of iterations increases. When the number of iterations *δ* > 40, the RMSE value does not change significantly. When *λ*_2_ = 0.2, the final RMSE value is the lowest.

**Figure 8:**
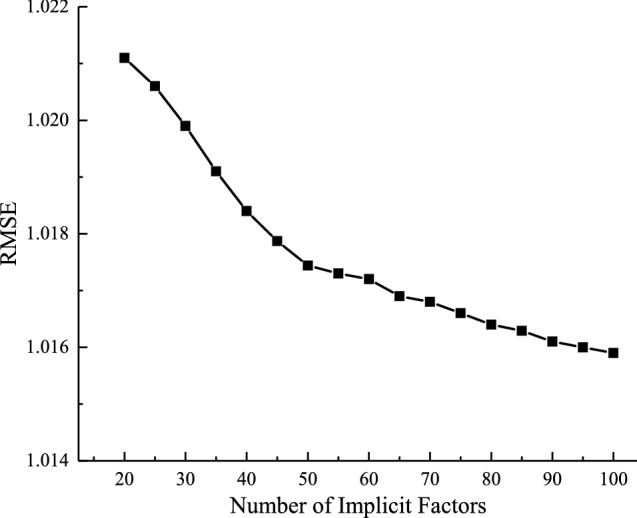
The influence of the number of latent factors *k* on the algorithm of this paper.

To analyze the effect of the number of latent factors *k* on the algorithm in this paper, let the learning rate *γ* = 0.007, the L1 regularization factor *λ*_1_ = 0.25, the L2 regularization factor *λ*_2_ = 0.2, and the iteration termination condition is RMSE ≤ 0.002, the results of latent factor optimization are shown in Fig. 8. As the number of latent factors *k* increases, the RMSE values become smaller and eventually level off.

Considering the RMSE and the complexity of the model, the parameters determined are: *γ* = 0.007, *λ*_1_ = 0.25, *λ*_2_ = 0.2, *k* = 75. At this point, RMSE = 1.016, MAE = 0.745. The algorithm has satisfactory recommendation accuracy, and the recommended coating matching results are verified in the test set.

To further evaluate the effectiveness of the proposed coating matching recommendation method based on improved fuzzy comprehensive evaluation of user labels and collaborative filtering algorithm. We compared the method proposed in this paper (IFCE-CF) with the fuzzy comprehensive evaluation and collaborative filtering based algorithm (FCE-CF) and the coating matching recommendation method based on direct scoring method and collaborative filtering by shipyard craft personnel (D-CF). Among them, the FCE-CF algorithm firstly constructs the coating matching evaluation system based on the fuzzy comprehensive evaluation method, and then carries out the recommendation of coating matching based on the collaborative filtering algorithm. The D-CF algorithm gets the coating matching score directly from the coating expert and then recommends the coating matching based on the collaborative filtering algorithm. The prediction results of different models are shown in Table 11.

**Table 11:** Model prediction performance comparison.

Model name	RMSE	MAE
D-CF	1.172	0.989
FCE-CF	1.064	0.834
IFCE-CF	1.016	0.745

As can be seen from the table, the RMSE and MAE of our proposed coating matching recommended method are significantly better than the other two methods. Its RMSE decreased by 0.156 compared to D-CF and MAE decreased by 0.089 compared to FCE-CF. The IFCE-CF method was verified to have higher prediction accuracy.

The hybrid algorithm of fuzzy comprehensive evaluation and collaborative filtering based on improved user labels is proposed in this paper. The index weights are constructed by combining user interest labels and hierarchical analysis, and a collaborative filtering algorithm based on matrix decomposition is used to achieve personalized recommendations for coating matching. It solves the current problem of over-reliance on technologist's experience in selecting coating matching in shipyards and realizes intelligent recommendation of ship coating matching. It can predict the shipowner's preference based on the data in the shipowner's historical orders and accurately recommend the coating matching in each painting area of the ship, providing a strong basis for the shipyard technologist to select the coating matching. At the same time, the method has important guiding significance and practical application value for the scientific management of the coating process and the quota calculation, selection and procurement and inventory management of coatings.

## Conclusion

This paper presents a coating matching solution recommendation method to help shipyard technologist to recommend unused coating matching to ship owners according to their different needs. A fuzzy comprehensive evaluation method combined with user label improvement is proposed. Taking into account various factors affecting the selection of coating matching, five main indexes are extracted and refined into 17 sub-indexes. The weights of the indexes are determined by combining the weights of user interest labels and hierarchical analysis, so as to achieve a comprehensive evaluation of the coating matching. Based on this, a collaborative filtering algorithm based on matrix decomposition is used to recommend the coating matching. The recommended method of coating matching proposed in this paper was tested based on the historical data of shipyard painting. The test results show that the prediction accuracy of this method is higher than the other methods mentioned in the paper, and its RMSE and MAE are significantly lower.

Due to the particularity of ship construction, the amount of data for the existing coating matching scores is limited, which leads to a certain gap between the accuracy of the recommended results and the ideal value. Therefore, building a more accurate recommendation model for coating matching based on small sample data is an important direction to be explored in the next step.
